# Genomewide Analysis of Carotenoid Cleavage Dioxygenases in Unicellular and Filamentous Cyanobacteria

**DOI:** 10.1155/2012/164690

**Published:** 2012-02-28

**Authors:** Hongli Cui, Yinchu Wang, Song Qin

**Affiliations:** ^1^The Coastal Zone Bio-Resource Laboratory, Yantai Institute of Coastal Zone Research, Chinese Academy of Sciences, Yantai 264003, China; ^2^Yantai Institute of Coastal Zone Research, Graduate University of the Chinese Academy of Sciences, Beijing 100049, China

## Abstract

Carotenoid cleavage dioxygenases (CCDs) are a group of enzymes that catalyze the oxidative cleavage steps from carotenoids to various carotenoid cleavage products. Some *ccd* genes have been identified and encoded enzymes functionally characterized in many higher plants, but little in cyanobacteria. We performed a comparative analysis of *ccd* sequences and explored their distribution, classification, phylogeny, evolution, and structure among 37 cyanobacteria. Totally 61 putative *ccd* sequences were identified, which are abundant in *Acaryochloris marina* MBIC 11017, filamentous N_2_-fixing cyanobacteria, and unicellular cyanobacterial *Cyanothece*. According to phylogenetic trees of 16S *rDNA* and CCD, *nced* and *ccd*8 genes occur later than the divergence of *ccd*7, *apco*, and *ccd*1. All CCD enzymes share conserved basic structure domains constituted by a single loop formed with seven *β*-strands and one helix. In this paper, a general framework of sequence-function-evolution connection for the *ccd* has been revealed, which may provide new insight for functional investigation.

## 1. Introduction

Cyanobacteria, also known as blue-green algae and blue-green bacteria, are among the earliest branching groups on earth, dating back 2.5–3.5 billion years, based on the fossil evidence [1]. They may be unicellular or filamentous and can be found in almost every conceivable environment, such as marine and freshwater habitats, soil, rocks, and plants [2, 3]. With the capacity of oxygenic photosynthesis similar to the process found in higher plants, cyanobacteria constitute a group of species diverse not only in ecological habitat, but also in genome size and the number of gene, indicating the significance of comparative genome research. The genome size varied from 1.6 Mb (*Prochlorococcus* sp. MIT9301) to 9.0 Mb (*Nostoc punctiforme* PCC 73102), and the number of gene ranged from 1,756 (*Prochlorococcus marinus* MED4) to 8,462 (*Acaryochloris marina* MBIC11017) [4–6].

**Figure 1:**
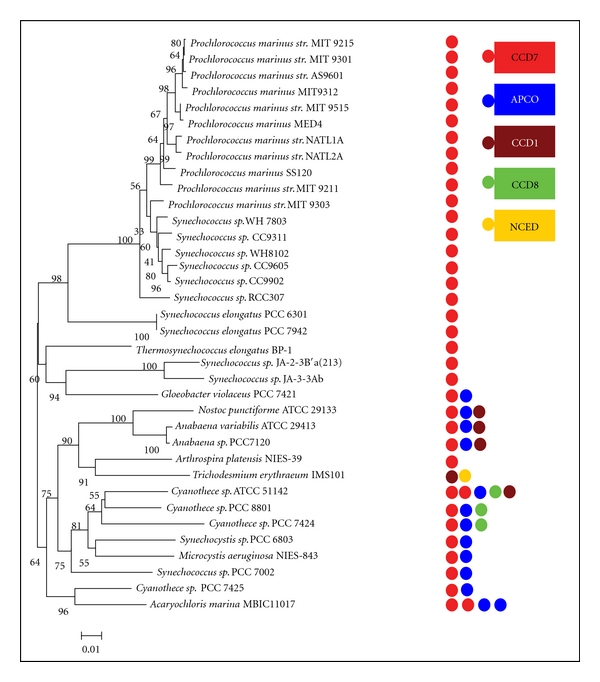
Phylogenetic tree of the sequenced cyanobacterial strains and the distribution of *ccd* sequences genes. Left: 37 fully sequenced cyanobacteria strains were performed based on 16 s *rRNA* as described in the methods section. An identical topology was obtained with two different methods (ML, NJ) and different models applied (for ML, generalized time reversible (GTR); for NJ, Kimura 2-parameter). *Numbers* at the node indicated bootstrap values (%) for 1,000 replicates. Right: the distribution of *ccd* genes across 37 cyanobacterial strains. Circular boxes represented the different *ccd* sequences that were labeled in the distinct color. The numbers of *ccd* sequences were presented by the numbers of circular boxes.

A lot of information on the evolutionary history of cyanobacteria has strongly supported an underlying meaning of comparative genome research. Three major clades are observed in cyanobacteria phylogenetic tree (Figure 1). The unicellular cyanobacteria (*Prochlorococcus* and *Synechococcus*) from ocean form the first monophyletic group (BS: 98%). They maintain the smallest genome size and account for significant biomass and primary production of marine biosphere [7]. Two *Synechococcus elongatus* PCC (6301 and 7942) are found at the base of this monophyletic group. Three thermophilic cyanobacteria (*Synechococcus* sp. JA-2-3B′a (2-13), *Thermosynechococcus elongatus* BP-1, and *Synechococcus* sp. JA-3-3Ab) and a thylakoid-lacking marine cyanobacterium (*Gloeobacter* sp. PCC 7421) [8] together compose the second monophyletic group (BS: 60%). In the last monophyletic group (BS: 64%), the filamentous N_2_-fixing cyanobacteria (*Nostoc* sp. PCC 7120, *A*. *variabilis* ATCC 29413, *N. punctiforme* ATCC 29133, *A*. *platensis* NIES-39, and *T. erythraeum* IMS101) build the sister group (BS: 75%) with freshwater unicellular cyanobacteria (*Cyanothece* sp. ATCC 51142, 8801, 7424, bloom forming* Synechocystis* sp. PCC 6803, and toxic bloom* Microcystis aeruginosa* NIES-843). An animal-cyanobacterial symbiont (*Acaryochloris marina* MBIC11017) and *Cyanothece* sp. PCC 7425 build the basal branch of this monophyletic group (see Figure 1 and Supplementary Material Table 1 available online at doi: 10.1155/2012/164690).

The first identified gene encoding carotenoid cleavage dioxygenase was the *Vp14* maize gene which was required for the formation of the abscisic acid (ABA) precursor xanthoxin, representing the rate-limiting step in ABA biosynthesis [9, 10]. Subsequently, homology-based analysis of the existence in all taxa was carried out *in silico*, which allowed the elucidation of the biosynthesis of several carotenoid-derived compounds, such as retinal in animals [11, 12] and fungi [13], the pigments bixin [14], saffron [15], and neurosporaxanthin [16]. On the basis of the identified substrates and presumed mechanism of catalysis, these enzymes are referred to as carotenoid cleavage dioxygenases (CCDs).

Carotenoid cleavage dioxygenases (CCDs) are a group of enzymes that catalyze the oxidative cleavage steps from carotenoids to various carotenoid cleavage products (apocarotenoids). These apocarotenoids function as signalling molecules with diverse functions including the ubiquitous chromophore retinal, plant hormone abscisic acid, and strigolactones [17, 18]. Other apocarotenoids with unknown functions in plants with high economic value are bixin in *Bixa orellana* and saffron in *Crocus sativus* [19]. These enzymes are present in all taxa [17, 20] and exhibit a high degree of regio- and stereospecificity for certain double bond positions as opposed to their frequent promiscuity towards substrates [21]. CCD enzymes have been widely studied in higher plants. In *Arabidopsis thaliana*, the CCD family includes nine members forming the basis for CCD classification (NCED, CCD1, CCD4, CCD7, and CCD8) in plants [22, 23]. The members of CCD family, *Cmccd4a*, *Cmccd4b*, *Cmnced3a*, and *Cmnced3b *have, been identified and functionally investigated in *Chrysanthemum morifolium *[24]. Among CCD family, NCED subfamily involves in abscisic acid (ABA) biosynthesis [10, 17, 23]. The majority of CCD has been shown to reside in plastids, whereas the only exception is CCD1 and its orthologous enzymes in higher plants, which act in the cytoplast to generate C13 and C14 apocarotenoids. Known pathways of carotenoid cleavage leading to various apocarotenoids have been discussed [17, 20, 21, 25, 26]. According to endosymbiotic theory, chloroplasts in plants and eukaryotic algae evolved from cyanobacterial ancestors *via *endosymbiosis.

It is reasonable to speculate that the CCD enzymes are widely separated in cyanobacteria as well. At present, many *ccd *genes have been cloned and functionally characterized *in vitro *from some sequenced cyanobacteria strains, including *Synechocystis *sp. PCC 6803 [27] and *Nostoc *sp. PCC 7120 [28–30]. With the completion of genome sequencing of several cyanobacterial species, modifications and supplements are needed.

Recently, 37 genomes of unicellular and filamentous cyanobacteria have been available, which facilitate cyanobacterial systemic analysis for metacaspases family [31], serine/threonine protein kinases [32], restriction modification system [33], and carotenoids biosynthesis [34]. These genome-sequencing projects undoubtedly bring great convenience to the searching for novel *ccd* genes by bioinformatic tools. In this study, five characterized *ccd *genes from *Arabidopsis thaliana *and *Zea mays *were selected as queries to search for cyanobacterial *ccd *genes. A BLASTP-plus-phylogeny reconstruction approach was employed to analyze CCD protein sequences in cyanobacteria, emphasizing an overall view on their distribution, classifications, phylogeny, evolution, and structure. Better understanding of cyanobacterial CCD enzymes may provide deeper insights into the evolution and the functional investigation of CCD enzymes in all organisms.

## 2. Materials and Methods

### 2.1. Computational Search for Novel ccd Genes

37 species of cyanobacteria, including *Prochlorococcus*, *Synechococcus*, *Synechocystis*, *Gloeobacter*, *Cyanothece*, *Microcystis*, *Trichodesmium*, *Acaryochloris*, *Anabaena,* and *Nostoc* (http://genome.kazusa.or.jp/cyanobase/), were used in this analysis. The *ccd* genes from higher plants *Arabidopsis thaliana*, *Tomato*, and *Zea may* (Table 1) were obtained from National Center for Biotechnology Information (NCBI) and were used to construct a query protein set. Each protein in this query data set was used to search for the potential novel sequences in all cyanobacterial species from whole genome sequences available, by using the basic local alignment search tool (BLASTP) with *E*-value < 1*E*−10 [35–37]. Proteins found by this method that fit the criteria for genuine CCD enzymes were added to the query set for another round of BLASTP searches. The searches were iterated until convergence.

### 2.2. Multiple Sequence Alignment and Phylogenetic Analysis

All protein sequences identified by BLASTP were aligned using ClustalW (http://www.ebi.ac.uk/Tools/msa/clustalw2/) [38]. The final alignment was further refined after excluding the relatively poorly conserved regions at the protein ends and consisted of sequences spanning the conserved domains. 16S *rDNA* and CCD phylogenetic trees were constructed by the NJ (Neighbor-Joining) method, using the program MEGA 4.0 (http://www.megasoftware.net/) [39] and ML (Maximum-Likelihood) method, using the program PHYML (http://www.atgc-montpellier.fr/phyml/binaries.php/) [40], with bootstrap support values deriving from 1000 randomized and replicated datasets. The Le and Gascuel evolutionary model [41] was selected for the protein phylogenies assuming an estimated proportion of invariant sites and a gamma correction. Graphical representation and edition of the phylogenetic tree were performed with TreeDyn (v198.3) [42]. In the phylogenetic analysis of CCD, CCD enzymes of fungi were used as outgroup to root the tree.

### 2.3. Motif Scanning and Structure Domain Analysis

To identify conserved motifs, Multiple Expectation Maximization for Motif Elicitation (MEME) version 2.2 [43] was employed with a set of parameters as follows: number of repetitions-any, maximum number of motifs-100, number of sites (≥5 and ≤50), and optimum motif width set to ≥6 and ≤200 [44, 45]. The Simple Modular Architecture Research Tool (SMART) (http://smart.embl-heidelberg.de/) and Conserved Domains Database (CDD) (http://www.ncbi.nlm.nih.gov/cdd/) were applied to predict the structure domains of these CCD proteins sequences, relying on hidden Markov models and Reverse Position-Specific BLAST separately [46, 47].

### 2.4. Tertiary Structure Prediction

To well understand the evolution of certain enzyme, protein structure was analyzed using homology modeling. The protein sequences of CCD from *Prochlorococcus marinus* MIT9312 (PMT9312_0282), *Nostoc punctiforme* ATCC 29133 (Npun_F0298), *Cyanothece* sp. PCC 7424 (PCC7424_5321), and *Anabaena* sp. PCC 7120 (all1106) were submitted to the protein model server: SWISS-MODEL Web server [48, 49] (http://swissmodel.expasy.org/) though Automated Model, respectively. All the manipulations were performed using Pdb-Viewer [50, 51].

## 3. Results

### 3.1. Identification of Putative CCD Proteins

The 37 completed cyanobacteria were used in this research, and the detail information about key features of these cyanobacterial species was summarized in Supplementary material Table 1. The phylogenetic tree based on 16S *rDNA* was showed in Figure 1. The candidate genes identified and the distribution across cyanobacterial strains in this study were listed in Table 2 and Figure 1. Totally 61 putative *ccd* genes were predicted and annotated from 37 completed cyanobacterial genomes using BLASTP programs with the query sequences. 50 of these genes were originally annotated as retinal pigment epithelial membrane protein (RPE), lignostilbene-**α**, **β**-dioxygenase (LSD), or carotenoid oxygenase (CO). The remaining 11 proteins were accepted as CCD enzymes in this research, including 5 hypothetical proteins, 4 undefined proteins, 1 apocarotenoid 15,15′-dioxygenase (APCO), and 1 similar to neoxanthin cleavage enzyme. Interestingly, RPE65 (retinal pigment epithelium 65-kDa protein) shows a significant degree of sequence homology to the CCD family. Thus, many CCD enzymes were originally annotated RPE in cyanobacteria.

Amid diverse cyanobacterial genomes, the number of *ccd* gene varies from 1 to 5. Within unicellular cyanobacteria, N_2_-fixing *Cyanothece* sp. ATCC 51142 has 5 *ccd* genes, much more than other species. An animal-cyanobacterial symbiont (*Acaryochloris marina* MBIC11017) has 4 *ccd* genes. All of the *Synechococcus* and *Prochlorococcus marinus* strains have only one *ccd* gene except that *Synechococcus sp. *PCC 700 has two* ccd *genes. Two unicellular strains inhabit in freshwater (*Synechocystis *sp. PCC 6803 and *Microcystis aeruginosa *NIES-843) and one marine cyanobacteria (*Gloeobacter violaceus *PCC 7421) each contains two *ccd *genes. The *Cyanothece* group has two or more *ccd* genes, including *Cyanothece* sp. PCC 7425 (2), *Cyanothece *sp. PCC 8801 (3), and *Cyanothece* sp. PCC 7424 (4). Compared to unicellular cyanobacteria, filamentous N_2_-fixing cyanobacteria have more *ccd* gene (3 for *Anabaena* sp. PCC 7120, *Anabaena variabilis* ATCC 29413, and *Nostoc punctiforme* ATCC 29133, 2 for* Trichodesmium erythraeum* IMS101). However, *Arthrospira platensis* NIES-39 contains only one *ccd* gene.

### 3.2. Phylogenetic Analysis of ccd Genes in Cyanobacteria

**Figure 2:**
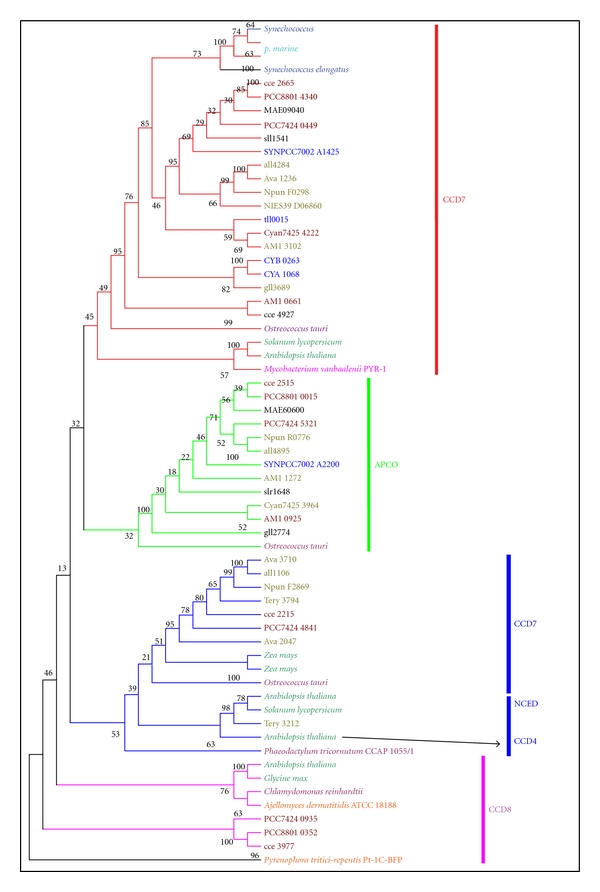
Maximum-likelihood tree of CCD enzymes of cyanobacteria, bacteria, eukaryotic algae, fungi, and higher plants. The model of LG+I+G was applied to construct ML tree using PHYML as described in the methods section. *Numbers* at the node indicated bootstrap values (%) for 1,000 replicates. Cyanobacterial CCD enzymes IDs and the strain names are as in Table 2 and Figure 1. Red line: CCD7, green: APCO, blue: CCD1, CCD4, and NCED, pink: CCD8. Species belonged to similar clade were represented by colored boxes. Orange: fungi, wheat: bacteria, purple: eukaryotic algae, barium: higher plants, cyan: cyanobacterial *Prochlorococcus marinus*, blue: *Thermosynechococcus elongates, Synechococcus elongates, and Synechococcus,* ruby: cyanobacterial *Cyanothece*, and smudge: filamentous cyanobacteria. Unlabeled species included *Synechocystis* sp. PCC 6803 (sll1541 and slr1648), *Gloeobacter violaceus* PCC 7421 (gll3689 and gll2774), *Microcystis aeruginosa* NIES-843 (MAE09040 and MAE60600).

Considering the confusion created by unspecific annotation with possibly separate evolutionary histories, the translated CCD proteins from cyanobacteria and the characterized CCD enzymes from bacteria, eukaryotic algae, higher plants, and fungi were used to perform a phylogenetic analysis. The tree topology matches our Neighbor-Joining tree (data not shown), and the high branch support values coincide with high neighbor joining bootstrap values, suggesting that the CCD phylogeny is robust in different tree reconstruction methods. Therefore, only the ML trees were displayed in this paper. The CCD phylogenetic tree was rooted in the CCD of fungi. Observation of the tree revealed that all CCD enzymes fell into four clades (Figure 2): clade 1: CCD7, clade 2: APCO, clade 3: CCD1/NCED/CCD4, and clade 4: CCD8.

As shown in Figures 1 and 2, the first clade, *ccd7*, was composed of almost all the cyanobacterial strains (in this paper), except for filamentous nitrogen fixation *Trichodesmium erythraeum* IMS101. The *ccd7*-homologous genes from two unicellular marine cyanobacteria *Synechococcus* (except for *Synechococcus* sp. PCC 7002) and *Prochlorococcus* constituted the first subfamily. Amino acid sequence identity of genes from this subfamily ranged from 59% to 98% (Supplementary Material Table 2). In the second subfamily, *ccd7*-homologous genes from two hot-spring habitat cyanobacteria (*Synechococcus* sp. JA-3-3Ab and *Synechococcus* sp. JA-2-3B′a (2-13)) and a thylakoid-lacking cyanobacterium (*Gloeobacter* sp. PCC 7421) clustered into a group. Other cyanobacterial *ccd7* genes clustered into the last group, including filamentous cyanobacteria and unicellular cyanobacterial *Cyanothece.* Moreover, *ccd7*-homologous genes were discovered among eukaryotic microalgae (*Ostreococcus tauri*), bacteria (*Mycobacterium vanbaalenii* PYR-1), and higher plants (*Solanum lycopersicum* and *Arabidopsis thaliana*). All of these CCD7s are orthologues because of obvious evolutionary relationships with high bootstrap value.

All the *apco*-homologous genes clustered into clade 2, including filamentous cyanobacteria (*Anabaena variabilis* ATCC 29413, *Anabaena* sp. PCC 7120, and *Acaryochloris marina* MBIC11017) and unicellular cyanobacteria *Cyanothece*. In addition, *apco*-homologous genes from *Gloeobacter violaceus* PCC 7421, *Microcystis aeruginosa* NIES-843, *Synechocystis* sp. PCC 6803, and *Synechococcus* sp. PCC 7002 were also assembled into this group. Amino acid sequence identity of genes from this clade ranged from 43% to 76% (Supplementary Material Table 3). It is interesting that two copies of *apco*-homologous genes existed in animal-cyanobacterial symbiont (*Acaryochloris marina* MBIC11017). Moreover, *ccd7*-homologous gene was discovered in eukaryotic algae (*Ostreococcus tauri*) while was absent in higher plants. Results from BLASTP and phylogenetic tree suggest that a close evolutionary relationship exists between CCD7 enzyme and APCO enzyme, between CCD7 enzyme and APCO enzyme.

CCD1 enzyme from filamentous cyanobacteria, algae, as well as higher plant (*Zea mays*) stays in a sister group relation with NCED and CCD4 enzyme from higher plant. They build the third monophyletic group with NCED enzymes from *Phaeodactylum tricornutum* as the basal branch of this clade. Amino acid sequence identity of genes from CCD1, NCED, and CCD8 was summarized in Supplementary Material Table 4. This group included all filamentous cyanobacteria, except for* A*. *platensis* NIES-39. Surprisingly, the *ccd1*-homologous gene was absent in all unicellular cyanobacteria except for *Cyanothece *ATCC51142, suggesting this organism might acquire this gene by horizontal gene transfer. It is interesting that the protein sequence of Tery_3212 from *Trichodesmium erythraeum *IMS101 is highly similar with NCED from *Arabidopsis thaliana *(99%). It is worth considering that CCD4 enzyme from *Arabidopsis thaliana *form a monophyletic with NCED enzyme, indicating that they may origin from common ancestor and acquire different function under natural selection during the evolution. CCD8 enzymes are widely distributed in eukaryote (fungi, eukaryotic algae, and higher plants), whereas they are missing in all cyanobacteria except for *Cyanothece *(PCC 7424, 8801 and ATCC 51142), suggesting these cyanobacteria obtain this gene by horizontal gene transfer or produce it later under the natural selection during the evolution.

### 3.3. Conserved Motifs

**Figure 3:**
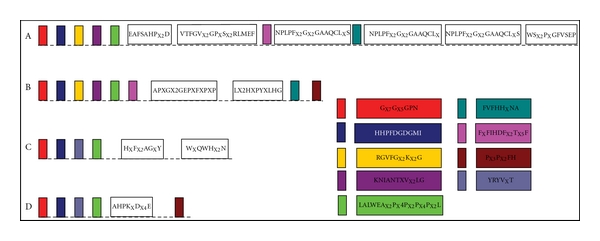
The conserved motifs of the cyanobacterial CCD enzymes. Schematic representation of motifs identified in cyanobacterial CCD enzymes using MEME motif search tool and ClustalW. The results were edited by hand. Length of box did not correspond to length of motif. Boxes represented the same or similar motifs that were labeled in the same color. (a) CCD7 from *Synechococcus* and *Prochlorococcus marinus*, (b) CCD7 from other cyanobacterial strains, (c) APCO, and (d) CCD1 and NCED.

According to CDD and SMART domain analyses, 8 protein sequences which were originally annotated as hypothesis protein or unidentified belonged to cyanobacterial CCD enzymes categories as well. All the protein sequences belonged to Pfam: RPE65 (IPR004294), which included *β*-carotene-15,15′-monooxygenase (BCDO1; EC 1.14.99.36), *β*-carotene-9′,10′-dioxygenase (BCDO2/CCD7), *9*-*cis*-epoxycarotenoid dioxygenase (NCED), *β*-apocarotenoid-15,15′-oxygenase (APCO), and retinal pigment epithelial membrane protein (RPE). However, domain analyses failed to further distinguish them. To facilitate the classification of different types of CCD enzymes, conserved motifs were identified by MEME tool and multiple sequence alignments with ClustalW. There were two typical glycine-rich (G_X7_G_X5_GP and HHPFDGDGMI) motifs and one leucine-rich (LALWEA_X2_P_X4_P_X2_P_X4_P_X2_L) motif existed in all cyanobacterial CCD proteins (Figure 3). Moreover, CCD7 from *Synechococcus *and *Prochlorococcus *organisms shared 59–98% similarities (Supplementary material Table 2) and possessed conserved patterns (EAFSAHP_X2_D, NPLPF_X2_G_X2_GAAQCL_X_S). CCD7 from other cyanobacteria organisms shared 54–95% similarities (Supplementary Material Table 5) and possessed conserved patterns (AP_X_G_X2_GEP_X_F_X_P_X_P and L_X2_H_X_PY_X_LHG). In addition, RG_X_FG_X_4GG, F_X_FIHDF_X2_T_X5_F, and FVFHH_X_NA motifs also existed in CCD7 enzymes from distinct organisms (Figures 3(a) and 3(b)). It was interesting that protein sequences of CCD7 and CCD1 have the same P_X3_P_X2_FH box in the N-terminal (Figures 3(b) and 3(d)). All these results suggested that all *ccd *genes have originated from a common ancestor and exhibited different functions under distinct natural selection during the evolution.

### 3.4. Structure of CCD Enzymes

**Figure 4:**
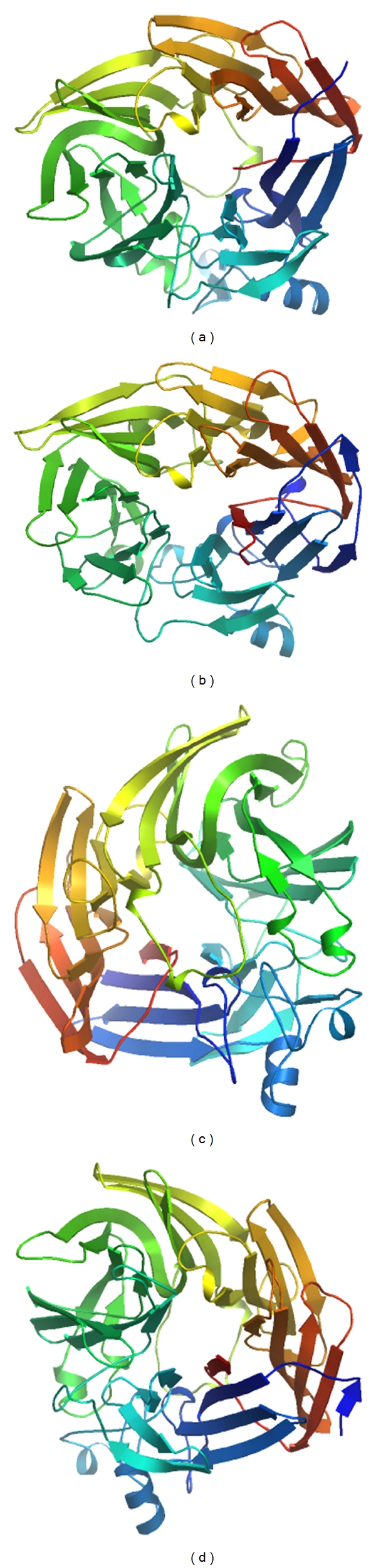
Model of structure of CCD enzymes from cyanobacteria strains. (a) The structure model of CCD7 from *Prochlorococcus* MIT9312 (PMT9312_0282), (b) the structure model of CCD7 from *Nostoc punctiforme* ATCC 29133 (Npun_F0298), (c) the structure model of APCO from *Cyanothece* sp. PCC 7424 (PCC7424_5321), (d) the structure model of CCD1 from *Anabaena* sp. PCC 7120 (all1106).

To understand the evolution of cyanobacterial CCD enzymes, protein structures of CCD7 from *Prochlorococcus *MIT9312 (PMT9312_0282) and *Nostoc punctiforme* ATCC 29133 (Npun_F0298), APCO from *Cyanothece* sp. PCC 7424 (PCC7424_5321), and CCD1 from *Anabaena* sp. PCC 7120 (all1106) were analyzed using homology modeling as described material and methods. A comparable analysis for the tertiary structure of different type of CCD-encoding ORFs from cyanobacteria revealed that a single loop formed with seven *β*-strands and one helix has conserved in four models, which may be related to binding domain (Figure 4).

**Figure 5:**
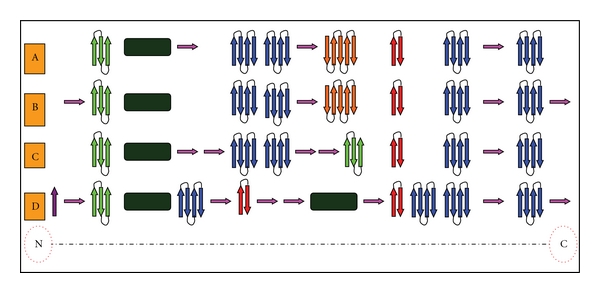
Sketch of the chain-fold of CCD enzyme showing the seven-bladed *β*-sheet as the basic motif. Distinct types of *β*-sheets were presented by colored arrowheads. Bottle green boxes stand for *α*-helix. (a) The second structure of CCD7 enzyme from *Prochlorococcus* MIT9312 (PMT9312_0282), (b) CCD7 from *Nostoc punctiforme* ATCC 29133 (Npun_F0298), (c) APCO from *Cyanothece* sp. PCC 7424 (PCC7424_5321), (d) CCD1 from *Anabaena* sp. PCC 7120 (all1106).

CCD7-encoding ORFs from different cyanobacterial organisms had conserved structure, which included **α**-helix (1), four-antiparallel *β* strand (4), two-, three-, and five-antiparallel *β* strand (1, 1, and 1), respectively. The additional sheets in the N- and C- terminal are the only difference (Figures 5(a) and 5(b)). As showed in Figures 5(c) and 5(d), five-antiparallel *β* strand existing in CCD7 was lacking in that of the APCO and CCD1. However, two *α*-helixes were present in CCD1-encoding ORFs. We supposed all the CCD-encoding ORFs in a given lineage may evolve through gene duplication that happened under natural selection during the evolution.

## 4. Discussion

The distribution of putative CCD encoding open reading frames (ORFs) in cyanobacteria is an integrated function of the genome sizes and the ecophysiological properties. In large extent, most cyanobacterial strains possess proportionate numbers of putative *ccd* genes with genome sizes, except for a few particular cases, for example, *Arthrospira platensis* NIES-39 and *Gloeobacter violaceu*s PCC 7421. All of* Prochlorococcus* and most *Synechococcus* (except for *Synechococcus* sp. PCC 7002) strains, which live in the oligotrophic open ocean and have smaller genome sizes, maintain one CCD encoding ORF. Gene duplication event or larger genome size than other *Synechococcus *species may be responsible for two *ccd* genes existed in *Synechococcus* sp. PCC 7002. Metacaspases were found to be absent in all *Prochlorococcus* and marine *Synechococcus* strains, except *Synechococcus* sp. PCC 7002 [31]. Compared to unicellular cyanobacteria with smaller genome sizes, CCD-encoding ORFs are abundant in filamentous with larger genome sizes. On the other hand, filamentous heterocystous cyanobacteria in response to the absence of combined nitrogen and exhibiting ecological properties including broad symbiotic competence with plants and fungi are responsible for containing more putative CCD-encoding ORFs even after allowing for their larger genome sizes [32, 33]. Moreover, according to our results, we speculated that the diverse distributions of *ccd* genes may reflect various environmental selective pressures. Unicellular *Cyanothece* that inhabits in freshwater has two or more CCD encoding ORFs, which is beyond the scaling effect of genome size. Similar phenomenon occurred in *Synechocystis* sp. PCC 6803 and *Microcystis aeruginosa* NIES-843. Considering the similar genome size, environmental selective pressure may take responsibility for this difference. This parallel pattern of distribution was provided by other cyanobacterial systemic analysis. For example, metacaspases systerm, Serine/threonine kinases, and restriction modification system in cyanobacteria indicate remarkable reduction of these proteins and environmental stress response systems in the ocean [31–33]. Gene lost is revealed to facilitate these cyanobacteria (*Prochlorococcus*) to acclimatize to the oligotrophic environment. The major driving force was supposed to be “a selective process sustaining the adaptation of these cyanobacteria,” which was discussed by Dufresne et al. (2005) in detail [52].

It is well accepted that there are NCED, CCD1, CCD4, CCD7, and CCD8 subfamily in higher plants [21]. Among them, NCED plays key role in cleavage of the 11,12 double bond of *9*-*cis* violaxanthin or *9′*-*cis* neoxanthin, which results in the formation of abscisic acid (C_15_) [10]. Our results showed that *nced*-homologous genes were absent in all cyanobacteria except for *Trichodesmium erythraeum* ISM 101, suggesting they did not contain ABA. These results were consistence with Pryce (1972), who supposed that, as distinct from higher plants, algae and liverworts did not contain ABA and its functions were fulfilled by lunularic acid [53]. Surprisingly, this hormone has been found in green microalgae (*Chlorella* sp., *Dunaliella salina*, and *Haematococcus pluvialis*) [54, 55] and also in the thalli of brown macrophytes from the genus *Ascophyllum* and some species of *Laminaria* [56].

Converting C_40_ trans-carotenoids to C_27_ apocarotenoids and the following step C_27_ to C_18_ is catalyzed by the CCD7 and CCD8 enzymes, respectively. They involve in the biosynthesis of strigolactones (C_18_) [57–59]. The recombinant CCD7 protein of *Arabidopsis* exhibited a specific 9′-10′(but not 9-10/9′-10′) cleavage activity *in vitro* converting *β*-carotene to the C_27_ compound *β*-apo-10′-carotenal and the C_13_ compound *β*-ionone. The C_18_ compound was formed by a secondary cleavage of the C_27_ apocarotenoid generated by AtCCD7 [59]. These two types of CCDs have transit peptides indicative of their action in plastids [60]. In this paper, *ccd7*-homologous genes were present in all cyanobacteria except for *Trichodesmium erythraeum* ISM 101 and *ccd8*-homologous genes were discovered in *Cyanothece*. Our results strongly support a cyanobacterial origin of CCD7 proteins in eukaryotic algae and higher plants (Figure 2). However, further investigation is necessary to explain whether a cyanobacterial origin of CCD8 proteins.

The formation of C_13_ and C_14_ from C_27_ is catalyzed by CCD1 or CCD4 enzymes in the higher plant [61–63]. The recombinant CCD1 or CCD4 enzymes from several plants have been shown to preferentially catalyze a single-step symmetrical cleavage at the 9-10/9′-10′ double bonds of various C_40_ carotenoids [17, 60, 64, 65]. Interestingly, the CsCCD4 recombinant enzymes produced considerably more **β**-ionone than CsCCD1, implying that the CCD4 enzymes may have higher enzyme activities [66]. In *planta*, CCD1 and CCD4 differ in their subcellular location being cytosolic and plastidial, respectively. Surprisingly, *ccd1*-homologous genes were discovered in N_2_-fixing cyanobacteria whereas *ccd4*-homologous gene was absent in all cyanobacteria (Figure 1). This relationship between the cyanobacterial, phototrophic eukaryotic algae, and higher plants strongly supports a cyanobacterial origin of CCD1 proteins (Figure 2). This conclusion has been demonstrated by Scherzinger and Al-Babili [29], who found that the NSC1 (NosCCD) enzyme is an ortholog of plant CCD1 enzymes [29]. Moreover, in *planta*, CCD1 and/or CCD4 might exhibit additional cleavage specificities including 7-8/7′-8′ cleavage in a certain subcellular environment or guided by a specific cofactor to provide the altered cleavage activity required for generation of crocetin and picrocrocin precursors. Recombinant CCD1 has been shown to exhibit 5-6/5′-6′ or 7-8/7′-8′ activities in addition to 9-10/9′-10′ under certain artificial conditions *in vitro* but only on acyclic or monocyclic substrates [67].

Apart from the numerous enzymes acting on C_40_ carotenoids, there are some special CCD enzymes converting only *β*-apocarotenals but not of bicyclic *β*-carotene. This has led to its classification as an apocarotenoid 15-15′-oxygenase (APCO). For example, APCO (apocarotenoid cleavage oxygenase) from the cyanobacterium *Synechocystis* sp. PCC 6803 cleaves the 15-15′double bond of various all-*trans*-apocarotenoids [20]. In this paper, *apco*-homologous genes were discovered by BLASTP analysis in some cyanobacteria and eukaryotic algae, which may be responsible for the production of apocarotenoids-derived volatile compounds in these organisms. For example, *Microcystis aeruginosa* blooms liberate *β*-ionone [20, 25]. Moreover, it was worthy consideration that *apco*-homologous genes were absent in higher plant and were evolutional closed with *ccd7* genes. Kiefer et al. [68] identified a new type of APCO, which catalyses the asymmetric cleavage of *β*-carotenoid at the C_9_′-C_10_′ position and produces the volatile compound **β**-ionone (C_13_) and apo-10′-carotenal (C_27_) [68].

Considering the sites (chloroplast) of C_40_ carotenoids biosynthesis and the location of each CCD enzyme, some hypothesis were summarized: (1) the additional function of cleavage C_40_ carotenoids suggests that the *ccd1* and *ccd7* genes may origin in cyanobacteria via eukaryotic algae towards higher plants; (2) the *ccd1 *gene transfers to cytosol by endosymbiotic gene transfer during the evolution in higher plants; (3) the CCD1, CCD7, and APCO enzymes occur earlier than other members of CCDs (CCD4, CCD8, and NCED) enzymes in cyanobacteria; (4) the* apco*-homologous gene was absent because of gene lost, whereas the *ccd4-*, *ccd8-*, and *nced*-homologous genes were present because of gene duplication in higher plant. These hypothesis were supported by one of our results that *ccd7*-, *apco-*, and *ccd1*-homologous genes were widespread in cyanobacterial strains, while *ccd8-* and *nced-*homologous genes only existed in some species, and *ccd4*-homologous genes were absent in all cyanobacteria (Figures 1 and 2).

Another interesting result was the highly similar tertiary structure for different types of CCD enzymes from distinct cyanobacterial strains (Figures 4 and 5). Indeed, sequence alignments within the CCD family clearly show that the most highly conserved regions are within the *β*-strands forming the propeller as demonstrated in Figures 3, 4, and 5. This strongly suggests that all members of the CCDs family have the same *β* propeller chain-fold and may therefore be modeled along the lines of the other CCD structures and share several same characteristics. Schwartz et al. [10], Redmond et al. [12], and Kiefer et al. [68] found that members of the CCD family share several characteristics: first, they require a Fe^2+^ for catalytic activity [10, 12, 68]; second, they contain four conserved histidines that are thought to coordinate iron binding; third, they contain a conserved peptide sequence at their carboxyl terminus that minimally constitutes a signature sequence for the family.

At present, the only established structure of a CCDs family member is apocarotenoids oxygenase (APCO) from *Synechocystis* PCC 6803 [69], and the mechanisms of choosing and modulating substrate specifically have been discussed [20, 70]. As observed in numerous *β* propeller structures, the active center of APCO is located near the propeller axis on the top side. The individual CCO family members choose and modulate substrate by changing length hand sequence of the connecting loops, while retaining the rigid propeller scaffold for stability [71–73]. More analysis is necessary to further delineate the mechanisms of choosing substrate exactly for each CCD family member. However, such an analysis is beyond the scope of this paper.

## 5. Conclusion

CCD enzymes play significant role in forming of carotenoids cleavage products, which are essential to several prokaryotes, eukaryotic algae, fungi, and higher plants. A total of 61 putative *ccd* genes have been identified across 37 species of cyanobacteria. The *ccd7*-, *ccd1*-, and *apco*-homologous genes are widespread in cyanobacteria and *ccd8*-homologous genes only exist in a few species. The distribution of *ccd* genes in unicellular and filamentous cyanobacteria relies on the genome size and ecological habitat. According to BLASTP results and phylogenetic tree of 16 s *rDNA* and CCD, it seems that CCD7, APCO, and CCD1 enzymes appeared earlier than other members of CCD enzymes. A slight difference exists between distinct types of CCD enzymes by motif scanning, while the secondary and tertiary structures are highly similar through homology modeling. All CCD enzymes share conserved basic configuration, which is constituted by a single loop formed with seven **β**-strands and one helix. This paper may provide new insight for the evolutional and functional investigation of CCD enzyme in cyanobacteria.

## Supplementary Material

Supplementary Table 1: List of kea features and genomes information from cyanobacterial strains analyzed in this paper. Note: HL, high light adaptation; LL, low light adaptation; GS, genome size.
Supplementary Table 2: The percentage of amino acid sequence similarity for CCD7 proteins from Synechococcus and Prochlorococcus.
Supplementary Table 3: The percentage of amino acid sequence similarity for APCO proteins from distinct organisms.
Supplementary Table 4: The percentage of amino acid sequence similarity for CCD1, NCED, and CCD8 proteins from distinct organisms.
Supplementary Table 5: The percentage of amino acid sequence similarity for CCD7 proteins from distinct organisms (except Synechococcus and Prochlorococcus)

## Figures and Tables

**Table 1:** Carotenoid cleavage dioxygenases protein sequences from distinct organisms in this paper.

Name	Species	Accession number	Type
NCED	*Arabidopsis thaliana*	NP_189064.1	*Streptophyta*
NCED	*Solanum lycopersicum*	CAB10168.1	*Streptophyta*
NCED	*Phaeodactylum tricornutum *CCAP 1055/1	XP_002177588.1	*Heterokontophyta*
CCD1	*Zea mays*	AAZ22348.1	*Streptophyta*
CCD4	*Arabidopsis thaliana*	sp|O49675.1	*Streptophyta*
CCD7	*Arabidopsis thaliana*	AEC10494.1	*Streptophyta*
CCD7	*Solanum lycopersicum*	ACY39882.1	*Streptophyta*
CCD8	*Arabidopsis thaliana*	NP_195007.2	*Streptophyta*
RPE	*Ostreococcus tauri*	XP_003080965.1	*Chlorophyta*
RPE	*Ajellomyces dermatitidis *ATCC 18188	EGE78156.1	Fungi
RPE	*Pyrenophora tritici-repentis *Pt-1C-BFP	EDU50284.1	Fungi
CO	*Mycobacterium vanbaalenii* PYR-1	YP_951059.1	Bacteria
PP	*Chlamydomonas reinhardtii*	EDP06596.1	*Chlorophyta*
LSD	*Ostreococcus tauri*	CAL53034.1	*Chlorophyta*
APCO	*Ostreococcus tauri*	CAL50095.1	*Chlorophyta*

**Table 2:** List of putative *ccd* genes identified across 37 cyanobacterial in this paper.

Species	Gene	Length	Annotation	Proposed function
*P. marinus *MED4	0280	507		CCD7
*P. marinus *MIT9312	0282	494	RPE	CCD7
*P. marinus *MIT9313	1879	507		CCD7
*P. marinus *SS120	0312	496		CCD7
*P. marinus str. *AS9601	03031	494	RPE	CCD7
*P. marinus str. *MIT 9211	03071	495	RPE	CCD7
*P. marinus str. *MIT 9215	03051	494	RPE	CCD7
*P. marinus str. *MIT 9301	03041	494	RPE	CCD7
*P. marinus str. *MIT 9303	25111	507	RPE	CCD7
*P. marinus str. *MIT 9515	03131	495	RPE	CCD7
*P. marinus str.* NATL1A	03601	497	RPE	CCD7
*P. marinus str. *NATL2A	1646	497	LSD	CCD7
*A. platensis *NIES-39	06860	492	LSD	CCD7
*S. elongates *PCC 6301	1315d	493	LSD	CCD7
*S. elongates *PCC 7942	0196	493	BCD	CCD7
*S. *sp. CC9311	0261	504	RPE	CCD7
*S. *sp. CC9605	0221	488	LSD	CCD7
*S. *sp. CC9902	0248	489	LSD	CCD7
*S. *sp. JA-2-3B′a (2-13)	0263	482	LSD	CCD7
*S. *sp. JA-3-3Ab	1068	482	LSD	CCD7
*S. *sp. PCC 7002	A1425	492	LSD	CCD7
*S. *sp. PCC 7002	A2200	484	RPE	APCO
*S. *sp. RCC307	2292	486	LSD	CCD7
*S. *sp. WH 7803	0270	495	LSD	CCD7
*S. *sp. WH8102	0227	489		CCD7
*S. *sp. PCC 6803	1541	490	HP	CCD7
*S. *sp. PCC 6803	1648	480	HP	APCO
*T. elongatus *BP-1	0015	487	LSD	CCD7
*T. *IMS101	3794	464	CO	CCD1
*T. *IMS101	3212	489	CO	NCED
*G. violaceus *PCC 7421	3689	482	LSD	CCD7
*G. violaceus *PCC 7421	2774	475	HP	APCO
*M. aeruginosa *NIES-843	09040	515	CO	CCD7
*M. aeruginosa *NIES-843	60600	490	HP	APCO
*N. punctiforme *ATCC 29133	2869	460	CO	CCD1
*N. punctiforme *ATCC 29133	0298	498	CO	CCD7
*N. punctiforme *ATCC 29133	0776	491	CO	APCO
*C. *sp. PCC 8801	0352	487	CO	CCD8
*C. *sp. PCC 8801	4340	495	CO	CCD7
*C. *sp. PCC 8801	0015	468	CO	APCO
*C. *sp. PCC 7424	4841	464	CO	CCD1
*C. *sp. PCC 7424	0935	488	CO	CCD8
*C. *sp. PCC 7424	0449	491	CO	CCD7
*C.* sp. PCC 7424	5321	477	CO	APCO
*C. *sp. PCC 7425	4222	501	CO	CCD7
*C. *sp. PCC 7425	3964	486	CO	APCO
*C. *sp. ATCC 51142	2215	459	RPE	CCD1
*C. *sp. ATCC 51142	3977	486	RPE	CCD8
*C. *sp. ATCC 51142	2665	494	LSD	CCD7
*C. *sp. ATCC 51142	4927	481	CO	CCD7
*C. *sp. ATCC 51142	2515	468	CO	APCO
*A. marina *MBIC11017	0661	488	LSD	CCD7
*A. marina *MBIC11017	3102	483	RPE	CCD7
*A. marina *MBIC11017	1272	479	RPE	APCO
*A. marina *MBIC11017	0925	485	RPE	APCO
*A. *sp. PCC 7120	1106	475	NCE	CCD1
*A.* sp. PCC 7120	4284	497	LSD	CCD7
*A. *sp. PCC 7120	4895	472	HP	APCO
*A. variabilis* ATCC 29413	2047	765	RPE	CCD1
*A. variabilis *ATCC 29413	3710	463	RPE	CCD1
*A. variabilis *ATCC 29413	1236	510	RPE	CCD7

## Abbreviations

CCD: Carotenoid cleavage dioxygenase
CCD (1, 4, 7, and 8): Carotenoid cleavage dioxygenase (1, 4, 7, and 8)
NCED: Nine-*cis*-epoxycarotenoid dioxygenase
APCO: Apocarotenoid 15,15′ oxygenase
RPE: Retinal pigment epithelial membrane protein
CO: Carotenoid oxygenase
LSD: Lignostilbene-**α**, *β*-dioxygenase
HP: Hypothetical proteins
UP: Undefined proteins
NCE: Neoxanthin cleavage enzyme
BS: Bootstrap value.
