# Supplementary material for: Genomewide Analysis of Carotenoid Cleavage Dioxygenases in Unicellular and Filamentous Cyanobacteria
Source: Comp Funct Genomics. 2012 Feb 28;2012:164690. doi: 10.1155/2012/164690 (PMC3299332; doi:10.1155/2012/164690)
Supplement: Supplementary file 1 — Supplementary Table 1: List of kea features and genomes information from cyanobacterial strains analyzed in this paper. Note: HL, high light adaptation; LL, low light adaptation; GS, genome size. Supplementary Table 2: The percentage of amino acid sequence similarity for CCD7 proteins from Synechococcus and Prochlorococcus. Supplementary Table 3: The percentage of amino acid sequence similarity for APCO proteins from distinct organisms. Supplementary Table 4: The percentage of amino acid sequence similarity for CCD1, NCED, and CCD8 proteins from distinct organisms. Supplementary Table 5: The percentage of amino acid sequence similarity for CCD7 proteins from distinct organisms (except Synechococcus and Prochlorococcus) [file 164690.f1.pdf]

Additional file 1- Table 1 Lists of kea features and genomes information from cyanobacterial strains analyzed in this paper. Note: HL, high light adaptation; LL, low light adaptation; GS, genome size

Species	Kea Features			GS (Mb)	Genes
T. elongatus BP-1	unicellular	hot-spring	thermophile	2.59	2,528
N. punctiforme ATCC 29133	filamentous	freshwater	nitrogen fixation	9.06	6,794
A. variabilis ATCC 29413	filamentous	freshwater	nitrogen fixation	7.07	5,724
A. sp. PCC 7120	filamentous	soil	nitrogen fixation	7.21	6,223
A. platensis NIES-39	filamentous		nitrogen fixation	6.79	6,676
T. erythraeum IMS101	filamentous	marine	nitrogen fixation	7.75	4,498
A. marina MBIC11017	unicellular			8.36	8,462
C. sp. PCC 7425	unicellular	freshwater	nitrogen fixation	5.79	5,380
C. sp. ATCC 51142	unicellular	freshwater	nitrogen fixation	5.46	5,359
C. sp. PCC 8801	unicellular	freshwater	nitrogen fixation	4.79	4,416
C. sp. PCC 7424	unicellular	freshwater	nitrogen fixation	6.55	5,763
M. aeruginosa NIES-843	unicellular	freshwater	toxic bloom	5.84	6,363
S. sp. PCC 7002	unicellular	marine		3.41	3,235
S. elongatus PCC 6301	unicellular	freshwater		2.75	2,580
S. elongatus PCC 7942	unicellular	freshwater		2.74	2,715
G. violaceus PCC 7421	unicellular	marine		4.66	4,484
S. sp. PCC 6803	unicellular	freshwater		3.96	3,725
S. sp. RCC307	unicellular	marine		2.22	2,583
S. sp. CC9311	unicellular	marine		2.61	2,944
S. sp. WH 7803	unicellular	marine		2.37	2,586
S. sp. WH8102	unicellular	marine		2.44	2,579
S. sp. CC9605	unicellular	marine		2.51	2,692
S. sp. CC9902	unicellular	marine		2.23	2,355
P. marinus str. MIT 9303	unicellular	marine	100m, LL	2.68	3,049
P. marinus MIT9313	unicellular	marine	135m, LL	2.41	2,326
P. marinus SS120	unicellular	marine	LL	1.75	1,928
P. marinus str. MIT 9211	unicellular	marine	83m, LL	1.67	1,900
P. marinus MED4	unicellular	marine	5m, HL	1.66	1,756
P. marinus MIT9312	unicellular	marine	132m, HL	1.71	2,007
P. marinus str. MIT 9215	unicellular	marine	HL	1.74	2,025
P. marinus str. MIT 9301	unicellular	marine	90m, HL	1.64	1,949
P. marinus str. AS9601	unicellular	marine	50m, HL	1.67	1,964
P. marinus str. MIT 9515	unicellular	marine	15m, HL	1.70	1,948
P. marinus str. NATL1A	unicellular	marine	30m, LL	1.86	2,236
P. marinus str. NATL2A	unicellular	marine	10m, LL	1.84	2,207
S. sp. JA-3-3Ab	unicellular	hot-spring		2.93	2,815
S. sp. JA-2-3B'a(2-13)	unicellular	hot-spring		3.05	2,914

Additional file 2 – Table 2 The percentage of amino acid sequence similarity for CCD7 proteins from *Synechococcus* and *Prochlorococcus*

	PMM0280	PMT9312_0282	PMT1879	Pro0312	A9601_03031	P9211_03071	P9215_03051	P9301_03041	P9303_25111	P9515_03131	NATL1_03601	PMN2A_1646	Syncc9605_0221	SYNW0227	Syncc9902_0248	SynRCC307_2292	SynWH7803_0270	sync_0261
PMM0280	100	97	61	61	96	64	96	97	61	85	65	65	60	61	60	62	62	61
PMT9312_0282		100	60	61	97	64	96	97	60	86	65	65	60	61	60	62	61	60
PMT1879			100	68	61	71	60	61	99	61	65	65	73	73	72	71	73	71
Pro0312				100	60	69	60	61	68	62	66	65	64	64	63	64	64	62
A9601_03031					100	64	94	98	60	86	66	66	59	60	59	62	61	61
P9211_03071						100	64	64	71	65	69	69	69	68	69	66	70	68
P9215_03051							100	95	60	85	65	65	60	61	60	62	61	61
P9301_03041								100	61	85	65	65	60	61	60	62	62	61
P9303_25111									100	60	65	64	73	73	72	71	73	70
P9515_03131										100	65	65	60	60	60	61	61	60
NATL1_03601											100	99	65	65	65	65	66	65
PMN2A_1646												100	65	65	65	64	66	65
Syncc9605_0221													100	94	90	75	76	75
SYNW0227														100	90	75	75	76
Syncc9902_0248															100	75	77	74
SynRCC307_2292																100	75	75
SynWH7803_0270																	100	82
sync_0261																		100

Additional file 3 – Table 3 The percentage of amino acid sequence similarity for ACO proteins from distinct organisms

	gll2774	slr1648	AM1_0925	Cyan7425_3964	AM1_1272	SYNPCC7002_A2200	all4895	Npun_R0776	PCC7424_5321	MAE60600	PCC8801_0015	cce_2515
gll2774	100	48	50	50	52	48	53	55	53	51	53	53
slr1648		100	43	47	48	43	46	49	48	45	48	45
AM1_0925			100	57	52	45	51	53	47	50	52	50
Cyan7425_3964				100	53	45	54	52	51	53	54	52
AM1_1272					100	46	54	55	54	49	52	50
SYNPCC7002_A2200						100	50	52	52	48	53	50
all4895							100	76	61	56	59	58
Npun_R0776								100	61	53	58	57
PCC7424_5321									100	55	64	61
MAE60600										100	61	60
PCC8801_0015											100	69
cce_2515												100

Additional file 4 – Table 4 The percentage of amino acid sequence similarity for CCD1, NCED, and CCD8 proteins from distinct organisms.

	**CCD8**	PCC8801_0352	cce_3977	PCC7424_0935	A. thaliana	C. reinhardtii	**NCED**	P. tricornutum	A. thaliana	S. lycopersicum	Tery_3212	A. thaliana	Z. mays	**CCD1**	O. tauri	Ava_2047	PCC7424_4841	cce_2215	Tery_3794	Npun_F286	all1106	Ava_3710
**CCD8**																						
PCC8801_0352		100	70	65	25	26																
cce_3977			100	64	23	24																
PCC7424_0935				100	25	25																
A. thaliana					100	33																
C. reinhardtii						100																
**NCED**																						
P. tricornutum								100	25	25	23	25	29		25	26	26	27	25	27	26	26
A. thaliana									100	60	99	39	40		29	34	32	33	34	34	32	33
S. lycopersicum										100	48	37	40		30	32	33	37	34	36	35	34
Tery_3212											100	39	31		27	32	29	29	28	29	27	29
A. thaliana												100	37		28	34	35	35	35	37	35	35
**CCD1**																						
Z. mays													100		31	40	36	44	40	44	44	44
O. tauri															100	34	34	36	35	32	33	32
Ava_2047																100	50	52	50	55	53	53
PCC7424_4841																	100	59	52	55	54	54
cce_2215																		100	56	64	59	59
Tery_3794																			100	60	57	58
Npun_F286																				100	74	74
all1106																					100	96
Ava_3710																						100

Additional file 5 – Table 5 the percentage of amino acid sequence similarity for CCD7 proteins from distinct organisms (except *Synechococcus* and *Prochlorococcus*)

	AM1_3102	Cyan7425_4222	all4284	Ava_1236	Npun_F0298	NIES39_D06860	SYNPCC7002_A1425	sll1541	PCC8801_4340	cce_2665	PCC7424_0449	MAE09040
AM1_3102	100	62	58	58	60	57	56	54	54	58	57	57
Cyan7425_4222		100	60	61	61	59	54	54	54	54	55	56
all4284			100	95	79	65	58	56	60	61	63	65
Ava_1236				100	79	65	59	56	60	61	63	64
Npun_F0298					100	62	58	55	61	61	60	63
NIES39_D06860						100	62	60	59	60	58	63
SYNPCC7002_A1425							100	59	57	62	62	62
sll1541								100	61	60	58	61
PCC8801_4340									100	69	63	62
cce_2665										100	66	64
PCC7424_0449											100	65
MAE09040												100
